# Is Sickle Cell Trait Really Innocent?

**DOI:** 10.4274/tjh.galenos.2020.2020.0344

**Published:** 2021-06-01

**Authors:** Mahmut Yeral, Can Boğa

**Affiliations:** 1 Başkent University Faculty of Medicine, Adana Dr. Turgut Noyan Training and Research Center, Clinic of Hematology, Adana, Turkey

**Keywords:** Sickle cell trait, Splenic infarct, Complication

## To the Editor,

Sickle cell trait (SCT) is seen in about 13.6% of the population living in the Mediterranean region of Turkey [1]. In areas where carriage is common, SCT is considered important for genetic counseling before marriage. Even carriers in widespread regions do not have sufficient information about SCT. Physicians, on the other hand, know that SCT may rarely cause serious complications, but this is often overlooked in daily practice. Is SCT as innocent as it is deemed to be? We explore this further with a clinical case.

After 8 h of exposure to cold-weather conditions below 0 °C, a 55-year-old man was admitted to the emergency department with severe abdominal pain. On physical examination, there was widespread guarding and rigidity. Laboratory examination results showed that he had a leukocyte level of 14,000/µL (70% neutrophils), hemoglobin level of 14 g/dL, and platelet level of 450,000/µL. Abdominal tomography images revealed widespread infarct areas in the spleen. Splenectomy had to be performed due to uncontrolled abdominal pain despite narcotic analgesics. At approximately 2 weeks after the splenectomy, the patient was readmitted with severe abdominal pain. Widespread thrombosis was detected in the portal vein. The patient was started on anticoagulant therapy. He lacked personal or family history of thrombosis and was investigated for myeloproliferative diseases and thrombophilia factors. Tests for JAK 2V617F and major BCR/ABL mutations were negative. A bone marrow examination revealed normal cellularity and the absence of fibrosis. Protein S, protein C, and antithrombin III values were within the normal reference ranges; tests for factor V Leiden, prothrombin gene mutations, and antinuclear antibodies were negative. A flow cytometric study performed with the FLAER method showed that the granulocytes and erythrocytes did not exhibit paroxysmal nocturnal hemoglobinuria. The Hb electrophoresis results were 38.7% for HbS, 2.9% for HbA2, and 58.4% for HbA. SCT, which is triggered by cold-weather conditions, had started the chain of complications.

In SCT, pathogenesis causing spleen infarction and other complications can triggered by factors such as dehydration, increased viscosity, high altitude, and temperature changes [2,3]. Hypoxia in the renal medulla increases sickling, leading to increased cytokines and microthrombi in capillaries and the vasa recta. Microscopic or macroscopic hematuria and abdominal pain develop after ischemia and necrosis. In addition to SCT-associated renal papillary necrosis, chronic kidney disorders and cases of renal medullary carcinoma with poor prognosis and metastasis have been reported [4,5].

There is some information in the literature on athletes and those working in severe conditions who experience exertion-related rhabdomyolysis and sudden death. To prevent serious complications, some countries have implemented national screening programs for newborns, soldiers, and individuals engaged in active sports [3,6]. Harmon et al. [7] reported a 37-fold higher risk of exertion death in football players with SCT than in their unaffected peers.

We think that a national newborn-screening program for diagnosing sickle cell disease should be implemented in regions where HbS carriers are common. The aim of the Hemoglobinopathy Control Program implemented in Turkey since 2003 is to provide genetic counseling to HbS carriers detected via premarital screening, direct them to prenatal diagnosis, and follow children with hemoglobinopathy after birth [8]. It should not be forgotten that SCT is not innocent. Individuals heterozygous for HbS should be informed about clinical problems caused by SCT and recommendations should be made. We believe that adequate information and counseling can minimize complications associated with SCT, such as morbidity and mortality. Screening of this risk group before certain professional or social situations can be life-saving for some carriers. These programs can create ethical and social problems; however, ethical problems in screening programs can be reduced if they are only used for training and information purposes, instead of being used to prevent people from carrying out certain activities.

Under the current conditions, screening programs, at least during prenatal genetic counseling prior to marriage, should give HbS carriers the opportunity to receive information from first-degree healthcare professionals and hematologists about SCT complications and prevention.
